# Analysis of college students’ canteen consumption by broad learning clustering: A case study in Guangdong Province, China

**DOI:** 10.1371/journal.pone.0276006

**Published:** 2022-10-13

**Authors:** Chun Yang, Hongwei Wen, Darui Jiang, Lijuan Xu, Shaoyong Hong

**Affiliations:** 1 School of Accounting, Guangzhou Huashang College, Guangzhou, China; 2 School of Data Science, Guangzhou Huashang College, Guangzhou, China; Asia University, TAIWAN

## Abstract

Investigation on college students’ consumption ability help classify them as from rich or relative poor family, thus to distinguish the students who are in urgent need for government’s economic support. As canteen consumption is the main part of the expenses of the college students, we proposed the adjusted K-means clustering methods for discrimination of the college students at different economic levels. To improve the discrimination accuracy, a broad learning network architecture was built up for extracting informative features from the students’ canteen consumption records. A fuzzy transformed technique was combined in the network architecture to extend the candidate range for identifying implicit informative variables from the single type of consumption data. Then, the broad learning network model is fully trained. We specially designed to train the network parameters in an iterative tuning mode, in order to find the precise properties that reflect the consumption characteristics. The selected feature variables are further delivered to establish the adjusted K-means clustering model. For the case study, the framework of combining the broad learning network with the adjusted K-means method was applied for the discrimination of the canteen consumption data of the college students in Guangdong province, China. Results show that the most optimal broad learning architecture is structured with 14 hidden nodes, the model training and testing results are appreciating. The results indicated that the framework was feasible to classify the students into different economic levels by analyzing their canteen consumption data, so that we are able to distinguish the students who are in need for financial aid.

## 1. Introduction

Consumption is a special behavior that reflects the people’s possession of objects. The possession of objective ability is a necessary condition for the implementation of behavior [1]. Consumption ability is a concept accumulated by the rapid development of the social economic system. For the consumption behaviors, in addition to the knowledge and skills of consumer action strategies, the identification, comparison and selection of products will take extra time and energy, while the price the products account for most of the causal ratio [2]. Awareness and attention about the well-being of people’s consumption ability and consuming behaviors have increased in many countries, including China. Consumers with more abundant disposable time and energy and sufficient economic conditions have stronger consumption power [3].

University is a microcosm of a society. College students are participating in the transition to independence on attention to a highlighted set of features from the social character [4]. College students’ consumption ability is the basis of social consumption behavior. When individuals do not have or have insufficient consumption ability, even if they have consumption intentions, they will eventually lack corresponding consumption behaviors. According to the results of the interviews, it is found that the individual’s lack of consumption connotation knowledge and action knowledge, economic ability constraints, individual inherent habits and preferences such as cynicism, lazy and troublesome personality barriers hinder consumer behavior [5]. Therefore, investigation on college students’ consumption property help classify them as from rich or relative poor family, thus to distinguish the students who are in urgent need for government’s economic support.

The habitual consumption behavior of the young adults in universities can reflect the economic level and the unbalanced development of the society in the next few years. College students’ consumption behavior is on concern. Eating consumption is recognized as universal consumption among many consumption methods [6, 7]. Thus the eating habits of the college students are the key factors that can directly reflect the consumption ability of the students’ families. Generally, the students’ eating habits in colleges and universities are mainly reflected in their canteen consumption records [8]. The characteristics of canteen consumption will release some prominent marker to tell the economic difference of the students’ families. Consequently, we can use the consumption characteristics of college students spending in the college canteen to classify and discriminate the students’ family economic situation.

In recent years, research on college students’ consumption ability has been actively carried out. For example, Kim et al. discussed the students’ behavior difference about clothing consumption in comparison between the clothing majors and non-majors [9]. Bruce et al. used conventional statistical methods (such as the p-value and some simple descriptive indicators) to study the male first-year college students’ consumption change on in sugar-sweetened beverage [10]. Gao et al. performed an empirical study on the mechanism of college students’ green consumption in China [11]. They used questionnaires and make statistical analysis, mainly accomplished by using the SPSS software and lack of novel design in methodologies. There are relatively few studies concerning on data mining by investigating machine learning methods for qualitative or quantitative analysis [12, 13]. A review article discussed the possibility of artificial intelligence technologies be analyzed for enhancing the educational systems for semantic web-based education [14]. Cloud computing technology was used to analyze the requirements of academic and administrative affairs, education affairs, and affiliated institutions in the colleges and universities [15]. However, in the era of big data, the consumption data is presented as large volume, low value density, diverse forms, and fast growth [16, 17]; machine learning strategy plays an important role in data analysis of the trends in college student consumption. More specifically, the students’ canteen data simply records the consumption amount at each time the students spend. The data is fragmented and dynamically increasing, which is bond to raise some challenging issues that probably hinder the computational model optimization [18]. Then some multi-variate statistical methods should be introduced into the design of the machine learning algorithmic flow.

Clustering is one of the important machine learning methods in many communities for data classification, discrimination, and pattern recognition [19]. Basically, it aims to group data points into different clusters based on their similarity or density [20]. In the past decades, many clustering algorithms have been proposed such as the K-means clustering [21], spectral clustering [22], min-max cut [23], and multi-view clustering [24]. A parallel fractional lion algorithm is recently reported for data analysis, the algorithm is tested on six standard databases [25], but it is not examined by practical collected data. The fact that it operates only based on the MapReduce framework limits its further application.

Among the existing clustering methods which are suitable for flexible application, the most popular one is the K-means clustering algorithm because its algorithmic flow is simple and efficient, and its operation only needs to learn a limited number of centroids to minimize the interclass data distances. However, the clustering performance of K-means is much related to its random initialization of the centroids [26]. Spectral clustering is able to prevent the uncertain interference of the randomness. It characterizes the data connection with an appropriate graph whose vertices represent the data points and the weights represent the connection between data pairs, and tries to partition the vertices into different clusters by minimizing the cut information [27]. For this consideration, we jointly perform spectral clustering in the K-means process, in the way of adjusting the spectral rotation to derive the underlying data connection. The adjusting process is detailly to perform eigen-decomposition on the graph Laplacian matrix transformed from the original data. The graph Laplacian matrix help to amplify the property of the data, and the eigen-decomposition is functional to extract the features [28]. After the adjusted operation, the normal K-means clustering is applied. The newly proposed methods is defined as adjusted K-means clustering.

Although the adjusted K-means method is advanced for classifying the students’ canteen consumption data, the data is raw and the feature information of the data is ambiguous. To this end, we design a broad learning neural network architecture to extract the feature variables that are rich in data information. Broad learning architecture is built up based on the broad learning system (BLS) established on the idea of a fully connected neural network (FCNN) accompanied with random vector functional link [29, 30]. Specifically, the input data is transformed into numbers of feature nodes by proper mapping functions, and then these features are concatenated together to generate a series of enhancement nodes randomly, which will be used to expand the network architecture in a broad sense. All the features nodes and the outputs of the enhancement nodes are connected together to feed into the output layer. The desired output weights are to be determined by a fast ridge regression of the pseudo-inverse of the system equation [31]. The weights and biases in BLS are all randomly generated, and further to be trained in data-driven manner. The incremental learning algorithm is incorporated into the BLS which makes the network can be remodeled fast in the broad expansion without a retraining process.

Based on the principle of BLS, the broad learning neural network (BLNN) architecture is constructed as a black box model in our study for the analysis of the college student’s canteen consumption. We designed the architecture to be trained in data iteration for optimal observation of the linkage weights. On the other hand, the BLNN training bias is not considered in the model training process, we designed a fuzzy-transformed strategy to catch the feature information of the data under the condition of a fuzzy interval range. What the fuzzy-transformed strategy does is to make a fault-tolerant mode to ensure a relative large amount of candidate feature variables for BLNN training. The fuzzy-transformation is in fusion design with the principal component analysis (PCA) method. Specifically, the data features were extracted by PCA algorithm before the data was input to the BLNN architecture, and the PCA procedure is improved by using the fuzzy rules of the triangular membership function. The fuzzy transformed PCA technique is able to inhibit the original noise interference and make the explicit characteristics of the principal components much remarkable [32].

We proposed an adjusted K-means clustering method for the discrimination of the college students based on their canteen consumption data, thus to classify the students into different economic levels. Also, a broad learning neural network is designed to extract the exact data features to improve the discriminant accuracy, and a fuzzy transformed PCA technique is employed to reduce the model uncertainty and thus to help reduce the classification errors. The remainder of this article is organized as follows. Section 2 introduced the adjusted clustering method, the BLNN architecture and the fuzzy transformed PCA technique as well as the specific algorithmic modification. Section 3 describes the data collection and present the descriptive statistical properties. Section 4 applies the fusion of the proposed methods to establish the combined discrimination models in a data-driven manner, to fulfil the model optimization, validation and evaluation. Section 5 makes conclusions.

## 2. Methodologies

### 2.1. The adjusted clustering algorithm

For unsupervised data analysis models, clustering analysis is the simplest classification method. Among the existing clustering methods, the most popular one is K-means clustering due to its good adaptiveness and high efficiency. It is used to tune the cluster centroids by each newly-added testing sample, to minimize the within cluster data distances [33].

The principle of K-means is understandable. Given a data matrix X = [*x*_1_, *x*_2_,…, *x*_*p*_] ∈ R^*d*×*p*^, the K-means clustering aims to partition X into *k* clusters ({*C*_*s*_|*s* = 1, 2, …, *k*}, 1 ≤ *k* ≤ *p*), to achieve that the within-cluster sum of squared distances can be minimized and the sum of squared distances between different clusters can be maximized. The objective function of K-means clustering is

$${\text{min}}_{\left\{C_{s}\right\}}\;\sum_{s=1}^{k}\sum_{x_{i}\in C_{s}}{\left\|x_{i}-\mu_{s}\right\|}^{2},$$ (1)

where ∥·∥ applies the Euclidean norm operation, *x*_*i*_ is the *i*-th sample in the *C*_*s*_ cluster set, *μ*_*s*_ is the centroid of *C*_*s*_. To optimize the K-means results, the membership of each data point and the centroid of each cluster are alternately updated.

However, the data available for clustering in this work is some designated statistical data which points to some specific properties of the raw data. In this point of view, we proposed an adjusted K-means algorithm as the improvement of the traditional K-means method, so that the cluster operation is much suitable to deal with the comprehensive statistic data. Given a built graph, we employ a two-step spectral clustering procedure. The first step is to perform eigen-decomposition on the graph Laplacian matrix to obtain the scaled cluster indicator matrix; The second step aims to make discretization based on the matrix, to get the final cluster assignment [28].

In the adjusted K-means details, we firstly construct a graph affinity matrix *A*_*p*×*p*_ according to certain similarity measures to depict the relationship in the data. Let *a*_*j*_ be the *j*-th vector of the matrix *A* = {*a*_*j*_|*j* = 1, 2 … *p*}, which corresponds to the cluster indicator vector for *x*_*i*_. Each element of vector *a*_*j*_ is defined as 0–1 binary values, 1 means that *x*_*i*_ ∈ *C*_*s*_, and 0 means otherwise. By defining the scaled cluster indicator matrix as *Y* = *A*(*A*^T^*A*)^−1/2^ whose column vectors are given by

$$y_{col}={\left[\underset{\sum_{j=1}^{col-1}p_{s}}{\underbrace{0,\ldots ,0}},\;\underset{p_{s}}{\underbrace{1,\ldots ,1}},\;\underset{\sum_{j=col+1}^{k}p_{s}}{\underbrace{0,\ldots ,0}}\right]}^{T}/\sqrt{p_{j}},$$ (2)

where *p*_*s*_ is the number of data in the *s*-th cluster. Then, the objective function of adjusted clustering can be formulated as

$${\text{min}}_{Y}\;Tr(Y^{T}LY),$$ (3)

where *L* is a kind of Laplacian matrix defined as *L* = *D* − *A*, in which *D* is a diagonal matrix with its *j*-th diagonal element calculated by the numerical summation of the vector *a*_*j*_. The operator Tr(∙) is to calculate the trace of the matrix.

Since *Y*^T^*Y* = (*A*^T^*A*)^−1/2^*A*^T^*A*(*A*^T^*A*)^−1/2^ = *I*, the embedding *Y* can be obtained by stacking the eigenvectors of *L* corresponding to its *k* smallest eigen values. However, *Y* is a real-valued matrix by default, then spatial rotation is necessary to perform discretization for the adjusted K-means clustering [34].

Suppose *R* is an arbitrary orthogonal matrix, for any solution *Y*, the matrix product *YR* is a new generated solution from *Y* by applying the corresponding spatial rotation. Therefore, spatial rotation aims at finding a proper orthogonal and normalized *R* such that the resultant *YR* is closer to the discrete indicator than *Y* in K-means. Mathematically, it aims to minimize the distance of *YR* and *Y*, namely,

$$\begin{array}{c} {\text{min}}_{A,R}{\left\|YR-A\right\|}^{2},\\ \text{s}.\text{t}.\;A\cdot 1_{k}=1_{p}\;\text{and}\;R^{\text{T}}R=I, \end{array}$$ (4)

where 1_*k*_ and 1_*p*_ are the all-one column vectors with sizes of *k* × 1 and *p* × 1, respectively. We can easily solve the function by using the alternative optimization method, therefore the final cluster assignment can be obtained. By the computation of formulae (2), (3) and (4), we can estimate that the adjusted K-means procedure is designed to run within an effort of O[*d*^6^ × *p*^2^].

### 2.2. The broad learning architecture

**Fig 1:**
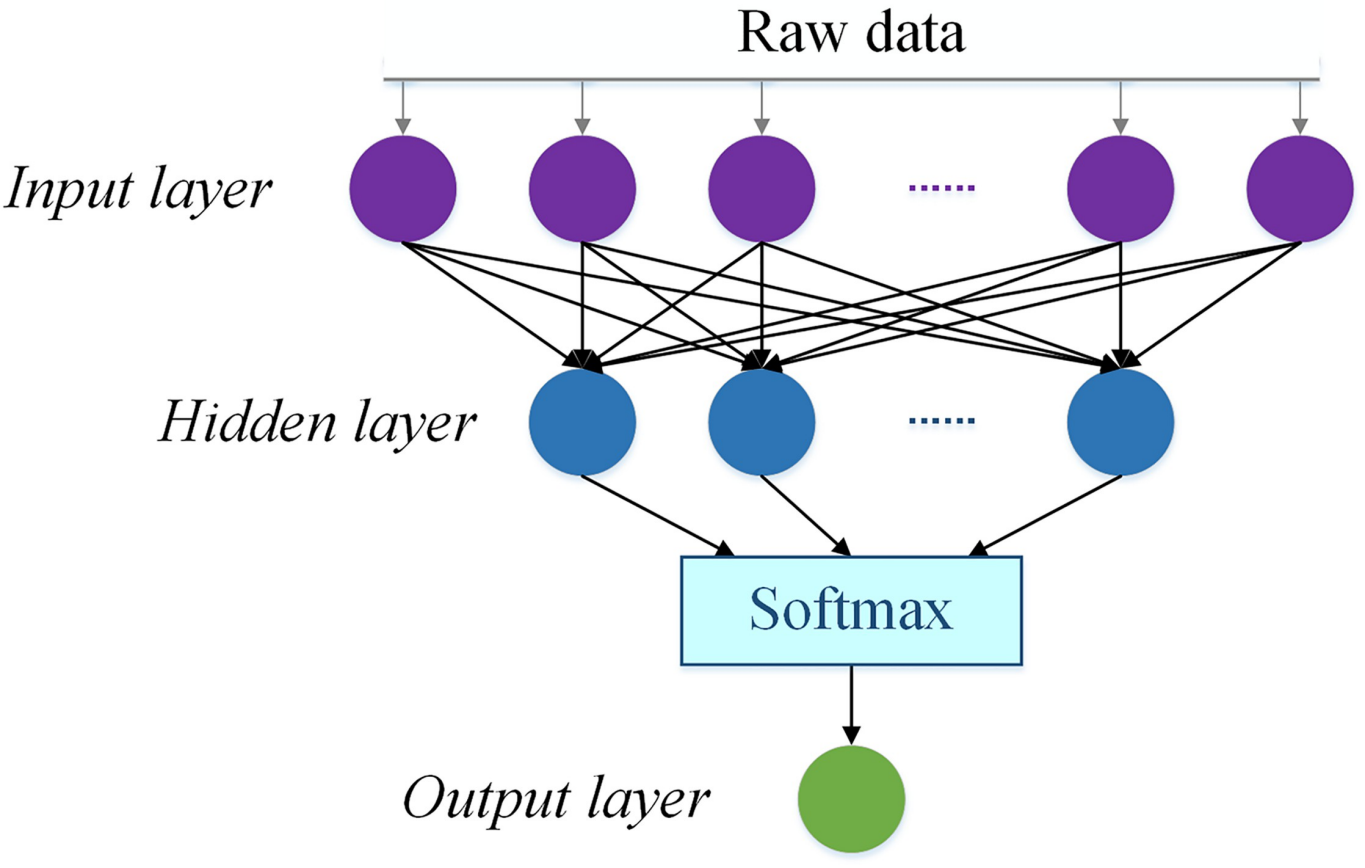
The FCNN structure.

The broad learning neural network architecture is built up on the basis of a fully connected neural network (FCNN). An FCNN model is for training an adaptive machine learning model by increase input of data information [35]. For instance, a basic single-hidden-layered FCNN structure is formed as shown in Fig 1. The original data ***x*** = (*x*_1_, …, *x*_*d*_)^T^ is taken as the input, delivered to the hidden layer with activation mapping on the linear summation of ***x***. Further the generated hidden variables are moved into a softmax unit, fitting a calibration model with multi-variate methods, to generated a discriminant/predictive results as the output data. The FCNN computation is formulated as

$$y=softmax\left(f_{2}\left(w_{2}^{T}\left(f_{1}\left(w_{1}^{T}x+b_{1}\right)+b_{2}\right)\right)\right),$$ (5)

where *f*_*ν*_ (*ν* = 1,2) is the activation functions, *w*_*ν*_ (*ν* = 1,2) is the linkage weights, and *b*_*ν*_ (*ν* = 1,2) is the constant thresholds. Subscript *ν* = 1 points to the calculation from the input layer to the hidden layer, *ν* = 2 means that from the hidden layer to the softmax unit, and finally to obtain the discriminant/predictive results in the output layer. On some loose definition, the softmax unit is taken as part of the output layer [36].

BLNN is an adaptive learning architecture designed based on the FCNN structure. It has similar data training function as a deep learning framework. BLNN has a widened pseudo input layer that can help to take one pre-clustering feature screening in one network structure. But in contrast to deep learning, broad learning is able to skip the process of long-time consuming on training abundant parameters in different filters and layers [29, 37]. The parameters in BLNN could be determined by random projection or by generating pseudo neural nodes to form a wide enough layer for fast tuning. Moreover, the training process can be changed to an incremental learning mode when the network structure is extended in the width direction [38]. Specific BLNN training procedure is showed as follows,

Considering the general supervised learning task, we are given the training dataset *X*_*p*×*d*_ containing *p* sample data of *d* properties, and the target *Y*_*p*×*k*_ marking the *p* samples as *k* discriminant classes. Each row data in *X* and *Y* denotes the original sample dimensional information and target clustered makers, respectively. The training samples are taken as the original input data with *d* properties.

Also, the input variables are applied to generate and connect the layer of enhancement nodes. To speed up the training process, the enhancement nodes are obtained in *N*_*a*_ groups. Any feasible algorithmic flow can be designed to observe the outputs of the groups of enhancement nodes.

Furthermore, the neurons in different enhancement layers are taken as added input nodes to widen the range of the input layer. The BLNN mechanism is to gather all of the enhancement nodes as well as the original input nodes, and flatten them as a brand new extended input layer.

$$X^{ext}=\left[X,T_{1},T_{2},\ldots ,T_{N_{a}}\right],$$ (6)

where *T*_*i*_ represents the *i*-th group of enhancement nodes obtained by a same transformation rule.

Thus the BLNN architecture is built up by re-constructing the FCNN with the new input data X^ext^, and the hidden variables are obtained in vector as

$$H=f_{1}\left(w_{1}^{T}X^{ext}+b_{1}\right),$$ (7)

where *H* = *H*_*p*×*m*_. The hidden variables are moved through the softmax unit and give discriminant/predictive results in the output layer, i.e.


$$\hat{Y}=softmax\left(f_{2}\left(w_{2}^{T}H+b_{2}\right)\right).$$ (8)

**Fig 2:**
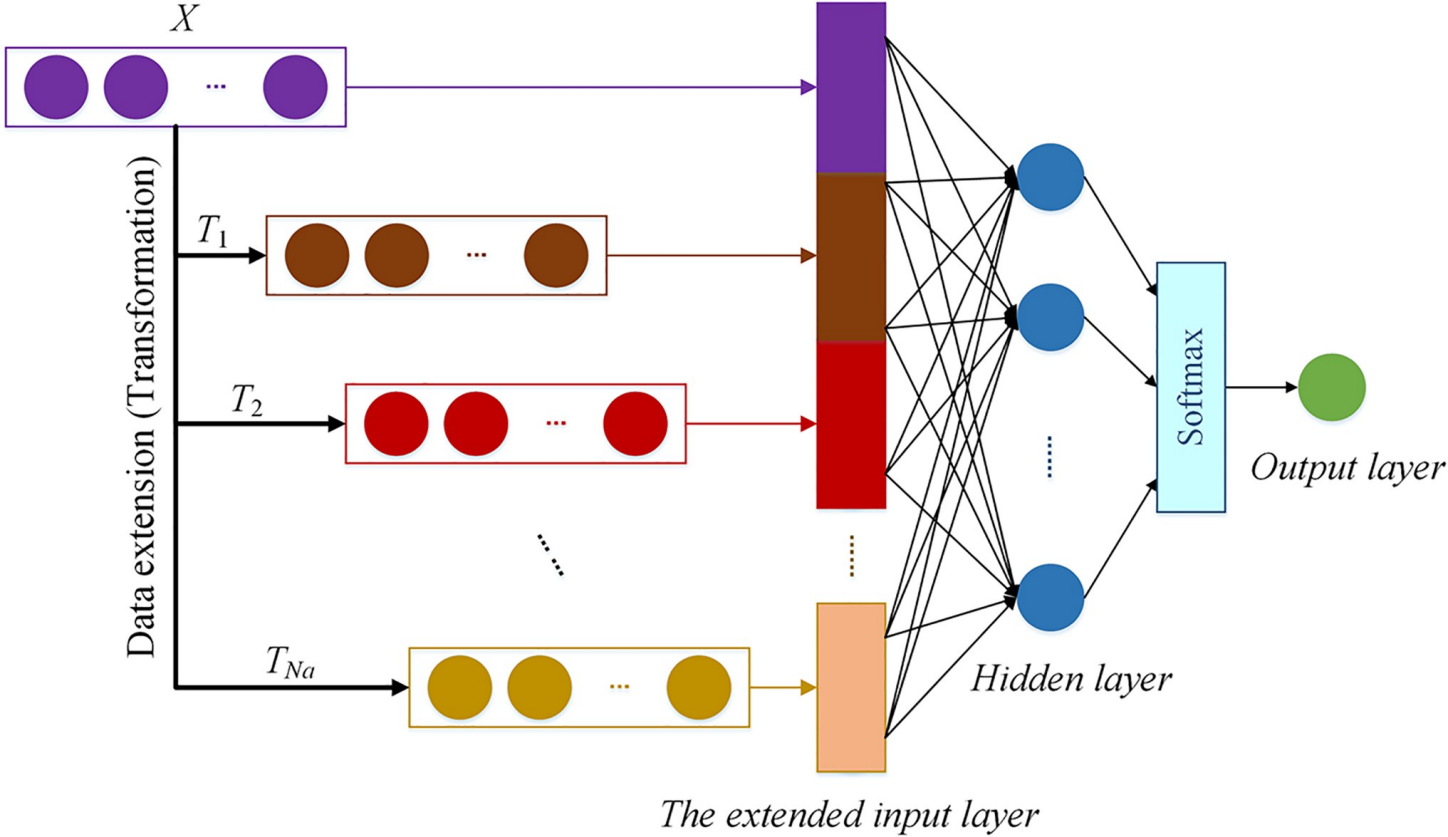
The BLNN architecture.

The BLNN architecture is shown in Fig 2. Practically, the BLNN architecture enables to test different number of hidden nodes (*m*), and adaptively tune the linkage weights (*w*_1_ and *w*_2_) for searching the optimal broad learning network structure. Before model training, we need to select suitable activation functions performing as *f*_1_ and *f*_2_ in advance. Concerning on the BLNN feature extraction by obeying the equations of (6), (7) and (8). The BLNN architecture runs in the complexity of O[*N*_*a*_^2^ × *d* × *p*^2^].

### 2.3. Fuzzy PCA transform technique

Fuzzy-transformed design is carried out under the condition of interval range of information data to be considered. It is used as the preprocessing algorithm for preventing the impact of the single type data [39]. It means to ensure that the fuzzy transform is functional to find the features from the consumption data that are not merely telling the consumption properties, then the model can be improved as conceiving a diversity of data characteristics. The fuzzy transform design of the classical PCA is able to emphasize the explicit informative features of the principal components so that the unnecessary noise can be suppressed.

**Fig 3:**
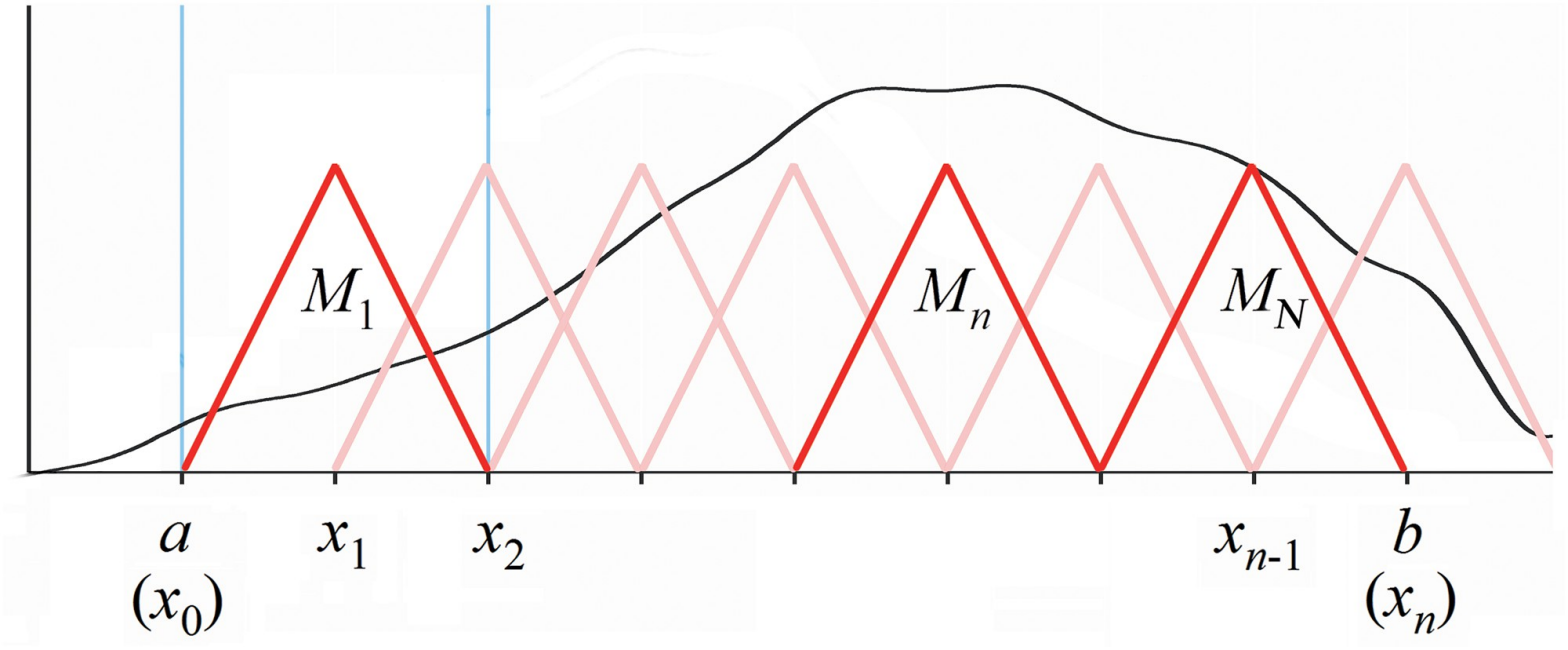
The triangular function for fuzzy transform.

The algorithm is proceeded by the fuzzy partition of the universe fuzzification. This depends on the feature contribution of the measured data. There are several membership functions can be used or modified to fit a specific target, such as the monotonic, triangular, trapezoidal and bell-shaped functions [40]. Specifically, the triangular function (see Fig 3) can much reduce the computational overhead of fuzzy transform due to simpler operation. The triangular fuzzy transform is briefly outlined as follows,

First to calculate the membership function *M*_*n*_,

$$M_{n}\left(x\right)\overset{\;k=1,2\ldots \left[\frac{n+1}{2}\right]\;}{⇐}\left\{\begin{array}{ll} M_{2k}\left(x\right)=1-\frac{\left\|x-x_{2k}\right\|}{\left\|c_{2k}\right\|}, & \text{for}\;\left\|x_{2k-1}\right\|<\left\|x\right\|<\left\|x_{2k}\right\|\\ M_{2k-1}\left(x\right)=\frac{\left\|x-x_{2k-1}\right\|}{\left\|c_{2k-1}\right\|}, & \text{for}\;\left\|x_{2k-2}\right\|<\left\|x\right\|<\left\|x_{2k-1}\right\|\\ 0, & otherwise \end{array}\right.$$ (9)

where the operator [·] is to get the floor integer; ∥·∥ applies the Euclidean norm operation; *x*_2*k*−1_ = {*x*_1_, *x*_3_, *x*_5_…} and *x*_2*k*_ = {*x*_2_, *x*_4_, *x*_6_…} are the original dataset; *c*_2*k*−1_ = {*c*_1_, *c*_3_, *c*_5_…} and *c*_2*k*_ = {*c*_2_, *c*_4_, *c*_6_…} are the principal components extracted by PCA.

Next to perform the fuzzy transform *F*_*n*_ of the *n*-th principal component.

$$F_{n}=\frac{\int_{a}^{b}f(x)M_{n}(x)dx}{\int_{a}^{b}M_{k}(x)dx},\;n=1,2\ldots N,$$ (10)

where the integral interval [*a*, *b*] piece-wise equals to [‖*x*_2*k*−1_‖, ‖*x*_2*k*_‖].

The fuzzy PCA transform algorithm is evident to generate a group of enhancement features in machine learning procedures [41]. Thus we designed to apply fuzzy PCA into the BLNN model to search for brand new network nodes, so that the data features are increasingly emphasized, but the computational complexity is increased by O[*N* × *k* × *p*].

## 3. Data collection and descriptive statistics

Experimental data was from colleges and universities located in Guangdong province, China. We collected a total of 307,968 students’ records from the year 2019 to 2021. The records include students’ personal information such as his/her age, majority and hometown. The properties of students’ consumption behaviors include card recharges, canteen expenses, utility bills, library borrowing, expenses for training and certification, etc. Commonly, every college student goes for meals in the college canteen, their expense on canteen meals is the key factor to tell their family backgrounds and economic status. Thus we targeted on the record of canteen expense data. There are some missing data and abnormal data in the 307,968 sample records. we removed them as they account for a small proportion in the total; then we have 265,972 samples for modeling analysis.

**Fig 4:**
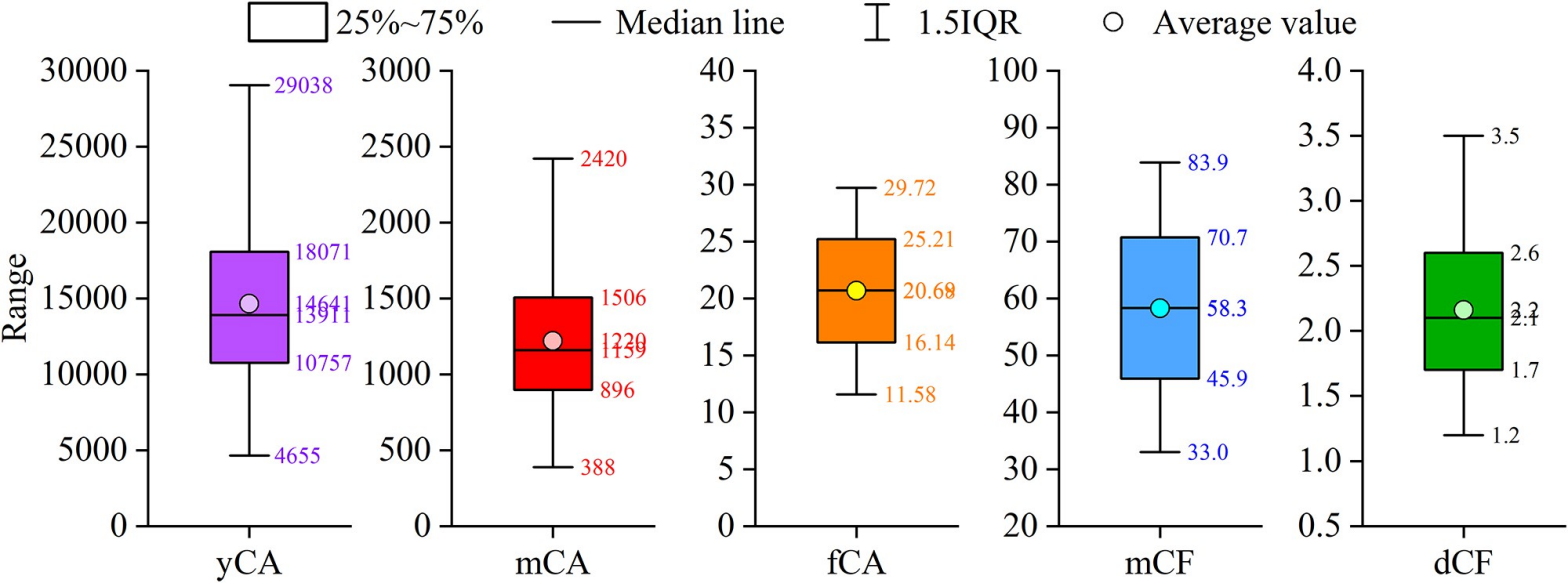
Distribution of the five quantified descriptive statistics.

In details, the canteen expense data is large and complex. It shows a long list of students’ consuming records in time orders. There appear heavy repeats of students’ personal information, the meal menus and the price. To reduce the influence of overlapping information, we design to use five common descriptive statistics to summarize the data features. They are the annual-averaged consumption cash amount (yCA), the month-averaged consumption cash amount (mCA), the averaged cash amount by consumption frequency (fCA), the month-averaged consumption frequency (mCF) and the day-averaged consumption frequency (dCF). There indicators are socially meaningful. A large value of yCA, mCA and fCA represents it is economically supported that the student is capable of enjoying good meals in canteen level. On the contrary, a small value of mCF and dCF means that the student is seldom expense on canteen meals, which indicates that he/she is economically supported to have high quality meals in good restaurants other than the college canteen. Fig 4 shows the distribution of the five quantified descriptive statistics.

**Fig 5:**
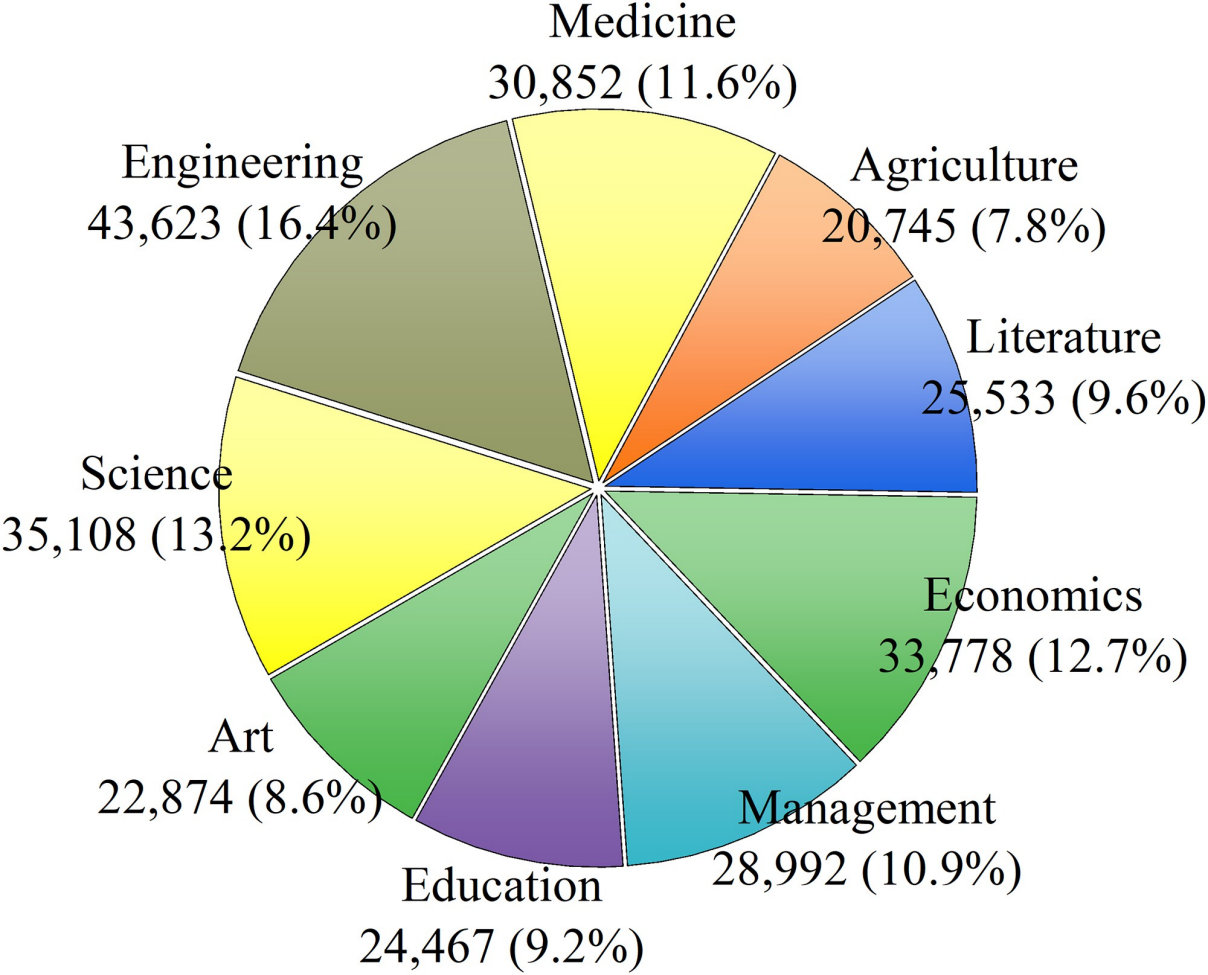
The distribution of students belonging to the 9 subject categories.

Additionally, some students’ personal information inherently influenced their consuming behaviors. On one hand, we surveyed the students’ majorities and summarized them into the subject category. The distribution by subjects is shown in Fig 5. There are relatively more students involved in the subjects of Engineering, Science, Economics, Medicine and Management, and relatively less students are in Agriculture, Art, Education and Literature. On the other hand, we also performed a statistical analysis of their hometown information. The 21 cities in Guangdong province are grouped into 7 regional segmentations according to their geographical locations. The numbers of students coming from each city group are listed in Table 1.

**Table 1:** The numbers and the percentages of students coming from each city group.

	Number of students	Percentage	In-group cities
Region 1	57,449	21.6%	Foshan, Guangzhou
Region 2	48,672	18.3%	Dongguan, Huizhou, Shenzhen
Region 3	40,961	15.4%	Jiangmen, Zhongshan, Zhuhai
Region 4	32,183	12.1%	Maoming, Yangjiang, Zhanjiang
Region 5	27,129	10.2%	Qingyuan, Yunfu, Zhaoqing
Region 6	28,993	10.9%	Heyuan, Meizhou, Shaoguan
Region 7	30,585	11.5%	Chaozhou, Jieyang, Shantou, Shanwei

## 4. Model designs and result discussions

### 4.1. Adjusted K-means modeling for discriminant metics

For all of the 265,972 targeting samples, we have the five important descriptive statistic indicators for modeling, i.e. yCA, mCA, fCA, mCF and dCF. The former three represent the consuming cash amount while the latter two are the consuming frequency. They are of different types and the data range vary a lot, thus we have to make a 0–1 normalization before modelling. Besides, the students’ consuming ability is certified by a large consuming cash amount reflected in yCA, mCA and fCA, and also by a small consuming frequency represented in mCF and dCF. As for modeling simplicity, we defined the consuming property vector as *v* = [yCA, mCA, fCA, 1/mCF, 1/dCF]^T^ for each target student sample, so that the sorting discrimination is totally uniform as from the smallest to the largest on each element. Then we applied the adjusted K-means method to the data for classification of the students’ consuming abilities.

**Fig 6:**
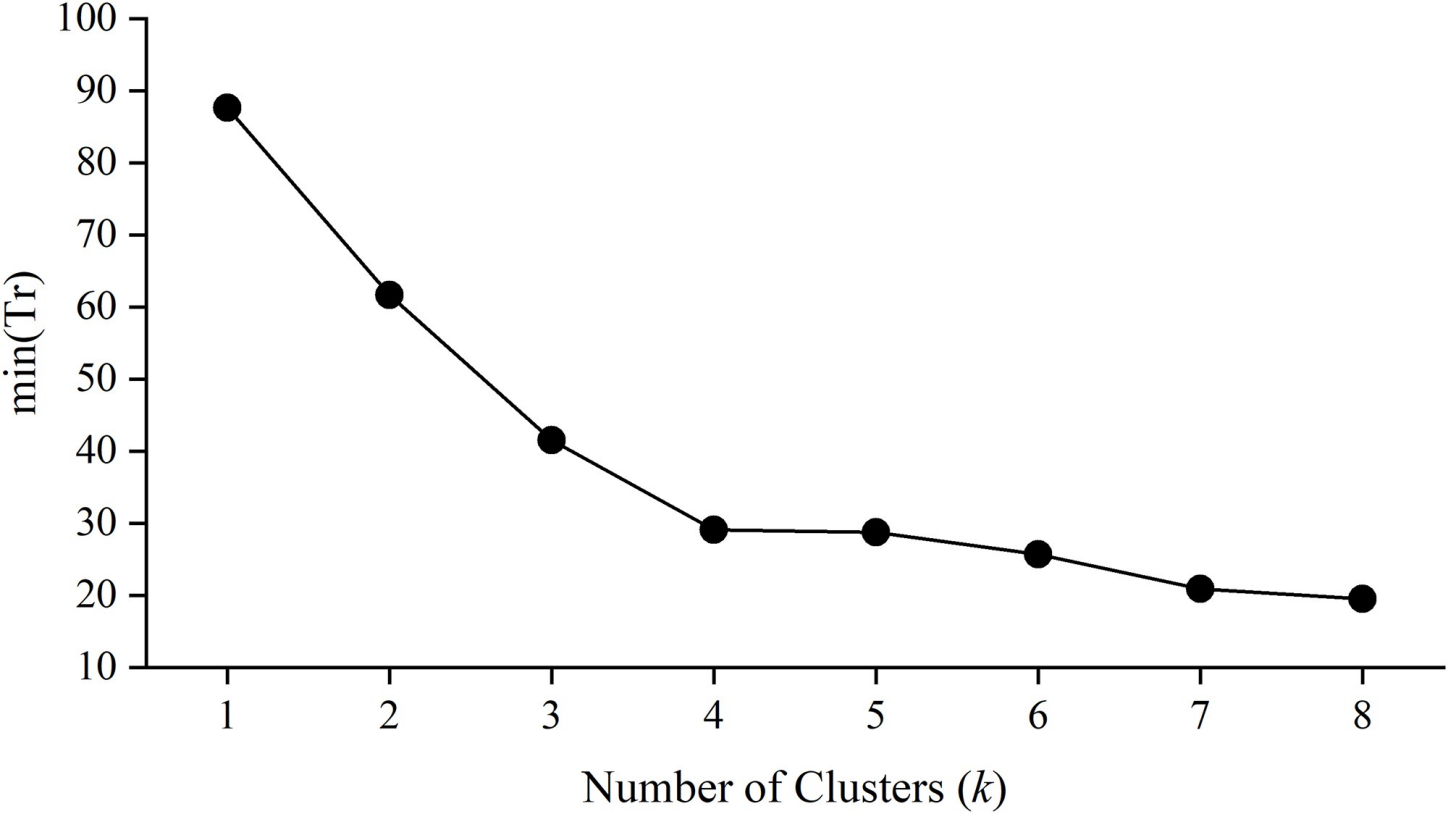
The elbow test for determination of the best number of clustering.

We used the elbow method to select the best number of clusters *k*. The elbow method defines a distortion quantified index on the objective function. a small value of distortion represents the samples clustered tightly, while a large value means the samples are loose [42]. We test several candidate *k* values for different mode of clustering, and find the objective function min *Tr* for each *k*. the comparative plot is shown in Fig 6. The curve indicated that *k* = 4 is the best choice before the clustering becoming loose. Therefore, we decide to classify the samples into 4 classes according to their consuming property indicators. The 4 classes are generated by the second norm value of the vector *v* (i.e. ‖*v*‖). The total of 265,972 samples are sorted in the ascending order of ‖*v*‖. To some extent, the sorting list is used to estimate the students’ consumption level. Then we can easily observe 4 piece-wise segments by dividing the minimum-to-maximum range by the points of Q1 (the 25% quantile), Q3 (the 75% quantile) and the median values. The segment from Minima to Q1 is marked as the cluster *C*_1_, the segment from Q1 to the Median is marked as the cluster *C*_2_, the segment from the Median to Q3 is marked as the cluster *C*_3_, and the segment from Q3 to the Maxima is marked as the cluster *C*_4_ (see Table 2). Thus we have the number of samples partitioned in each cluster set. The students classified in *C*_1_, *C*_2_, *C*_3_ and *C*_4_ are regarded as poor, frugal, normal and affluent, respectively, in the analysis of college canteen consuming behavior. The classification markers are further used to train and evaluate the discrimination models established by BLNN architecture.

**Table 2:** The definition of the 4 clustering classes by the quantile data segmentation.

	Segment 1	Segment 2	Segment 3	Segment 4
∥*v*∥	∈ [Minima, Q1]	∈ [Q1, Median]	∈ [Median, Q3]	∈ [Q3, Maxima]
Clustering class	*C*_1_	*C*_2_	*C*_3_	*C*_4_
No. of samples	49,562	75,519	83,757	57,134
Regarded as	Poor	Frugal	Normal	Affluent

### 4.2. Data extended by fuzzy PCA technique

Of the total of 265,972 samples, 186,180 samples (~70%) are used to train the model and 79,792 samples (~30%) used for model test. The test samples are not involved in the model training process. The training sample data *X* is used as the raw input data for BLNN training. The raw data is extended to produce one enhancement layer by using the fuzzy transform PCA technique, and then the enhancement neural nodes and the raw input data nodes are flattened as a novel extended input layer for broad network learning.

Concerning the raw data, the network architecture generates 5 neural nodes for receiving the 5-dimension normalized property variables [yCA, mCA, fCA, 1/mCF, 1/dCF]^T^. The 5-dimension property variables were globally converted into common principal components (PCs) sorted in the order of descending contribution rate. Then the PCs were further carried out for the fuzzy transform strategy, in which we used the triangular membership function. There we tuning the number of PCs from 1 to 5, to determine the best number of PCs for fuzzy optimization. The generated variables acquired by fuzzy transformed PCA was used to train the FCNN model. With adaptively searching the network linkage weights, appreciate discriminant results were obtained based on the186,180 training samples. There we observed the best FCNN model with aided transformation by the fuzzy-transform PCA technique. The optimal discrimination model was obtained by using 5 PCs, and the confusion matrix of the training samples was showed in Table 3. We have the discrimination accuracy as 89.9% from the confusion matrix. Accordingly, we set 5 extended enhancement neural nodes to build up the BLNN network architecture.

**Table 3:** The discriminant confusion matrix conducted based on the optimal FCNN classification model trained with the fuzzy–PCA–transformed 5 PCs.

		Classification by FCNN model with fuzzy PCA transform				Summation by available marker
		*C*_1_	*C*_2_	*C*_3_	*C*_4_	
Available marker	*C*_1_	**30,713**	1,027	859	2,094	34,693
	*C*_2_	1,733	**47,651**	1,962	1,517	52,863
	*C*_3_	1,532	1,794	**53,873**	1,431	58,630
	*C*_4_	1,274	1,596	1,977	**35,147**	39,994
Summation by classification model		35,252	52,068	58,671	40,189	


### 4.3. Data training by BLNN architecture

The BLNN training model is established by using the above-introduced BLNN architecture. We construct the raw input layer as having 5 neural nodes, for correspondence to receive the training sample data *X* which have 5 different properties. By testing different number of fuzzy PCs in Section 4.2, we finally decide to use 5 different transformed variables as the enhancement neural nodes for broad learning. The newly generated 5 PCs were included in one group denoted as *T*_1_ = {PC_1_, PC_2_, PC_3_, PC_4_, PC_5_}. Then the BLNN model is driven by flattening the original 5 input neurons (*X*) and the extended 5 enhancement neurons (*T*_1_) into a novel broadened layer. The broaden layer is taken as the refreshed input layer for network training. Accordingly, the original data *X* are extended to be *X*^ext^, which have a total of 10 different variables as follows,

$$X^{ext}={\left[X,\;T_{1}\right]}^{T}={\left[yCA,\;mCA,\;fCA,1/mCF,1/dCF,{PC}_{1},{PC}_{2},{PC}_{3},{PC}_{4},{PC}_{5}\right]}^{T}$$

The refreshed input layer is await for the data input of *X*^ext^. Then *X*^ext^ was delivered to the hidden layer by the algorithmic flow introduced in Section 2.2, generating a series of hidden nodes by activation function mapping of the summation of the input variables. The ReLU function [43] was applied for the hidden layer activation. The linkage weights (*w*_1_) was adaptively trained for iterative optimization, while the number of hidden nodes (*m*) is preset for tuning in the integer range of *m* ∈ [1, 2 … 16] ⊂ Z^+^.

**Fig 7:**
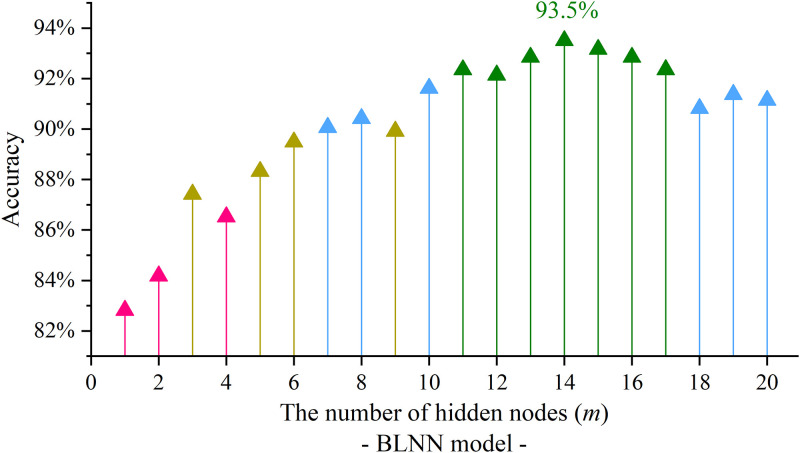
The tuning of the number of hidden neuron nodes for BLNN training.

Next, the hidden variables are delivered to the output layer by the similar operation of variable summation and activation function mapping, where we used the Sigmoid function [44] for output activation. The linkage weights (*w*_2_) was also set for adaptively iterative training. Afterwards, the output variables were involved in the softmax unit, and we used the adjusted K-means method to deduce the discriminant results. As for parameter tuning, different number of hidden nodes released different BLNN training results. We compared observations of model discrimination accuracy for using a changing value of *m* (see Fig 7). It is seen from the figure that we observed the best discrimination accuracy as 93.5% when *m* = 14. This indicated that the BLNN structure is best optimized by using 14 hidden nodes. Besides, the network structures built up with the numbers of from 11 to 17 hidden nodes are able to reach nearly appreciating discrimination results (see the green points in Fig 7). Their discrimination accuracies are also over 92%. For the comparison to the FCNN model, the discriminant confusion matrix of the optimal BLNN model was showed in Table 4. Although the BLNN model is able to improve the total accuracy, we can see in details that the better results are noticeable for the *C*_1_ class and the *C*_2_ class, while the *C*_3_ and *C*_4_ classes are not so obvious improved. The reasons are probably instinct to the segments for dividing the minimum-to-maximum range by the Q1, Q3 and the median values. The *C*_1_ and *C*_2_ classes are regarded as the poor and frugal level; It means that the students who consumes less in college canteen are relatively more easy to discriminated by the BLNN model. In contrast, the normal and the affluent level students (i.e. the *C*_3_ and *C*_4_ classes) can be classified to an equal extend by using the BLNN or using the FCNN model.

**Table 4:** The discriminant confusion matrix conducted based on the adaptively optimized BLNN model built up with 14 hidden neural nodes.

		Classification optimized BLNN model with 14 hidden neurons				Summation by available marker
		*C*_1_	*C*_2_	*C*_3_	*C*_4_	
Available marker	*C*_1_	**32,174**	726	621	1,172	34,693
	*C*_2_	1,134	**49,424**	1,486	819	52,863
	*C*_3_	988	1,209	**55,346**	1,087	58,630
	*C*_4_	669	951	1,229	**37,145**	39,994
Summation by classification model		34,965	52,310	58,682	40,223	


### 4.4. Model evaluation

There are 79,792 samples (~30% of the total) reserved before model training, which are excluded and totally independent from the model training process. The test samples were utilized to evaluate the best BLNN training model, as well as the FCNN model for comparison.

The optimized BLNN is built up with 5 original input nodes and 5 pseudo input nodes for broad learning, thus the extended input layer contains 10 neurons, for accepting the raw data variables (yCA, mCA, fCA, 1/mCF, dCF) and the generated comprehensive variables (PC_1_, PC_2_, PC_3_, PC_4_, PC_5_) transformed by the fuzzy PCA technique. The optimal network was identified as constructed with 14 hidden nodes by the abovementioned training fulfillment. The linkage weights were automatically tuning by the network adaptive training mechanism. The ReLU function was used as the activation functions for each neural perceptron. The softmax unit was designed to launch the adjusted K-means clustering algorithm for data classification.

**Fig 8:**
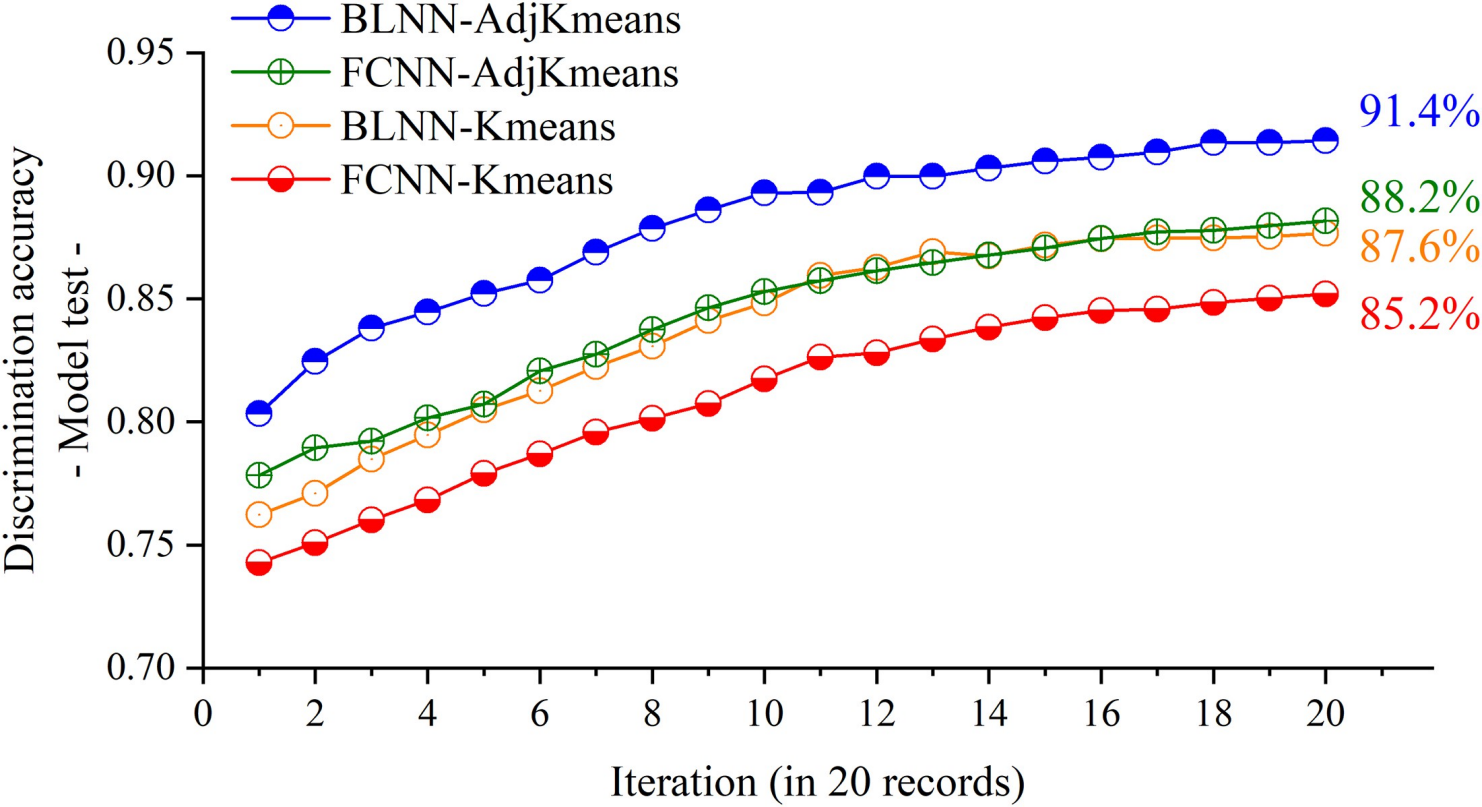
The 20 iterative records of the model improvement for the optimized BLNN–AdjKmeans model (blue) and the FCNN–AdjKmeans model (green), in comparison to the BLNN–Kmeans (yellow) and FCNN–Kmeans models (red). (Note that the label AdjKmeans represents the adjusted K–means method).

The data of the 79,792 test samples were input to test the optimized BLNN model. As the model training process is only to identify the optimal network structure, thus the model evaluation needs to re-train the network linkage weights for any new input. We recorded 20 of the changing classification accuracies during the adaptive iteration on the network linkage weights (showed in Fig 8), and the iterative discrimination results of the FCNN model were also noted in the figure for comparison. Fig 8 shows that the BLNN model observed the best discrimination accuracy of 91.4% for the test set samples. In contrast, the FCNN model was not able to reach up to 90%, its highest data only reached 88.2%.

As for comparison, the BLNN and FCNN extracted data features were delivered to establish the common K-means classification model. results were also illustrated in Fig 8. The comparative results indicated that the proposed BLNN architecture combined with the adjusted K-means clustering is functional to improve the model discrimination accuracy in data analysis for the classification of college student’s canteen consumption levels.

## 5. Conclusions

A broad learning clustering architecture was built up in this paper for the qualitative analysis of college students’ canteen consumption data. The fuzzy transformed PCA technique was applied to find extended data features before the data was input to the network. the adjusted K-means clustering method was fused in the network softmax unit for optimizing the classification model. Confronting the data of Guangdong college students, the BLNN structure is constructed by an extended input layer, a node-number-tuning hidden layer and an adaptive clustering softmax. The extended input layer was formed by a set of pseudo neural nodes for fast tuning. There identified 10 neural nodes, of which the former 5 are to receive the 5 descriptive statistical features of the raw data, and the latter 5 pseudo nodes are available for taking the 5 fuzzy PCs generated by fuzzy PCA transform technique, in which a triangle membership function was applied. Then the extended input data was trained through the hidden layer, and delivered to a softmax unit for establishing the adjusted K-means clustering models, thus to analyze the students’ canteen consumption data. The set-up BLNN modeling architecture is applied to classify the college students into 5 different economic level, by analyzing their canteen consumption data.

As to find the most optimal BLNN model for the discrimination, the hidden layer of the BLNN architecture was to tune the number of hidden nodes, and to test the model discrimination accuracy. The most optimal BLNN training model is structured as having 14 hidden nodes, the corresponding best training discrimination accuracy is high up to 93.5%. except for the best model, the BLNN models built up with from 11 to 17 hidden nodes were also able to get appreciating discrimination results over 92%. After that, the optimal training model was evaluated by using the test set samples. By 20 iteration model tuning, we observed the best testing discrimination accuracy as 91.4% by the BLNN model, which is better than the conventional FCNN model.

These modeling and comparing results indicated that the designed BLNN architecture is reasonable to get a more accurate discrimination results for the statistical evaluation of college students’ economic level, by analyzing the students’ canteen consumption recorded data. The design of BLNN framework in fusion use of fuzzy transformed PCA technique and the adjusted K-means method is an advanced machine learning mode. It is feasible to enhance the model effects for classifying college students into different economic levels, and thus we are able to make more accurate distinguishment of the students who are in need for financial aid. Further, the framework is prospectively expected to be applied in solving some challenging data mining issues in other fields.

## Supporting information

S1 Appendix (DOCX)
