# Supplementary material for: Analysis of college students’ canteen consumption by broad learning clustering: A case study in Guangdong Province, China
Source: PLoS One. 2022 Oct 13;17(10):e0276006. doi: 10.1371/journal.pone.0276006 (PMC9560066; doi:10.1371/journal.pone.0276006)
Supplement: S1 Appendix — (DOCX) [file pone.0276006.s001.docx]

# Appendix

**(I)** List of Acronyms

| **Acronym** | **Explanations** |
| --- | --- |
| BLS | Broad learning system |
| FCNN | Fully connected neural network |
| BLNN | Broad learning neural network |
| PCA | Principal component analysis |
| yCA | The annual-averaged consumption cash amount |
| mCA | The month-averaged consumption cash amount |
| fCA | The averaged cash amount by consumption frequency |
| mCF | The month-averaged consumption frequency |
| dCF | The day-averaged consumption frequency |

**(II)** List of Notations

| **Notation** | **Explanations** |
| --- | --- |
| $p$ | Number of samples |
| *d* | Number of properties |
| $X$ | The raw data matrix |
| $k$ | Number of clusters |
| $C_{s}$ | Clusters |
| $s$ | The subscript pointing to with cluster |
| $\left\Vert\cdot\right\Vert$ | Euclidean norm operator |
| $\mu_{s}$ | The centroid of $C_{s}$ |
| $A=A_{p\times p}$ | The graph affinity matrix |
| $a_{j}$ | The *j*-th vector of the matrix $A_{p\times p}$ |
| $Y$ | The scaled cluster indicator matrix |
| $p_{s}$ | The number of data in the *s*-th cluster |
| $L$ | The Laplacian matrix, defined as $L=D-A$ |
| $D$ | A diagonal matrix |
| $\mathrm{Tr}(\cdot)$ | The operator to calculate the trace of a matrix |
| $I$ | The identity matrix |
| $R$ | An orthogonal and normalized matrix for decomposition |
| $1_{k}$ / $1_{p}$ | All-one column vectors with sizes of $k\times1$ / $p\times1$ |
| $w_{\nu}$ | The network linkage weights |
| $b_{\nu}$ | The constant thresholds |
| $f_{\nu}$ | The activation functions |
| $\nu=1,2$ | The subscript pointing to the calculation between different layers. |
| $X^{\mathrm{ext}}$ | A brand new extended data |
| $N_{a}$ | The number of groups of the enhancement nodes in BLNN |
| $T_{i}$ | The *i*-th group of enhancement nodes in BLNN |
| *m* | The number of hidden nodes in BLNN |
| $H=H_{p\times m}$ | The variable vector in the BLNN hidden layer |
| $\hat{Y}$ | The feature variable vector in the BLNN output layer |
| $M_{n}$ | The membership function used in the fuzzy PCA technique |
| $\left[ \cdot\right]$ | The operator to get the floor integer |
| $c_{2k-1}$ / $c_{2k}$ | the principal components extracted by PCA |
| $N$ | The number of fuzzy transformed principle components |
| $F_{n}$ | the fuzzy transform of the *n*-th principal component |
